# Diverse Clinical Signs of Ocular Involvement in Cat Scratch Disease

**DOI:** 10.4274/tjo.28009

**Published:** 2017-01-17

**Authors:** Merih Oray, Sumru Önal, Aylin Koç Akbay, İlknur Tuğal Tutkun

**Affiliations:** 1 İstanbul University İstanbul Faculty of Medicine, Department of Ophthalmology, İstanbul, Turkey; 2 Koç University Faculty of Medicine, Department of Ophthalmology; VKV American Hospital, Ophthalmology Clinic, İstanbul, Turkey; 3 Koç University Faculty of Medicine, Department of Ophthalmology, İstanbul, Turkey

**Keywords:** Cat scratch disease, neuroretinitis, retinal infiltrate, optic neuropathy, endophthalmitis

## Abstract

**Objectives:**

To describe ocular manifestations, diagnosis, and treatment of cat scratch disease.

**Materials and Methods:**

Clinical records of patients with ocular cat scratch disease were reviewed.

**Results:**

Thirteen eyes of 10 patients (7 female, 3 male) with a mean age of 26.9±18.5 years were included. Nine patients had a history of cat contact and had systemic symptoms associated with cat scratch disease 2-90 days prior to the ocular symptoms. Ocular signs were: neuroretinitis in 4 eyes (associated with serous retinal detachment in the inferior quadrant in 1 eye), optic neuropathy in 2 eyes (1 papillitis and optic disc infiltration, 1 optic neuritis), retinal infiltrates in 6 eyes, retinochoroiditis in 1 eye, branch retinal arteriolar occlusion in 3 eyes, and endophthalmitis in 1 eye. Visual acuities at presentation were 1.0 in 7 eyes, 0.3 in 1 eye, ≤0.1 in 4 eyes, and light perception in 1 eye. Bartonella henselae immunoglobulin (Ig) M and/or IgG were positive in all patients. Systemic antibiotic therapy was administered in all patients. Systemic corticosteroid treatment (15-40 mg/day) was added to the therapy in 4 patients, following 5 days of intravenous pulse methylprednisolone in 2 patients. Treatment was ongoing for 1 patient and the mean treatment duration of the other 9 patients was 47±14.5 days. Visual acuities at final visit were 1.0 in 9 eyes, 0.8 in 1 eye, 0.4 in 1 eye, and no light perception in 1 eye.

**Conclusion:**

Cat scratch disease may present with different ocular signs and should be considered in the differential diagnosis in patients with such presentations.

## INTRODUCTION

Cat scratch disease (CSD) is a systemic condition caused by the gram-negative zoonotic bacillus *Bartonella henselae*.^1^ The disease is usually transmitted to humans via the scratch or bite of cats, its natural reservoir. Recently, the cat flea (*Ctenocephalides felis*) has also been implicated as an arthropod vector of the disease.^2,3^ The most common clinical manifestation is lymphoid CSD. An individual infected as a result of cat scratch or bite develops erythematous papules or pustules at the site of primary cutaneous inoculation. Within 1-2 weeks, patients develop regional lymphadenopathy (LAP) as well as flu-like systemic symptoms such as fever and fatigue. This stage of the disease is usually self-limited, resolving within a few weeks. LAP is usually unilateral and may affect a single lymph node in 50% of cases, multiple lymph nodes in 20% and multiple lymph node regions in 30%. LAP may be painful and suppurative. Headache, anorexia, nausea, vomiting, and sore throat may also occur. Patients may develop nonspecific maculopapular rash and erythema nodosum.^4,5^

Rarely, CSD may follow a disseminated course. The eye is the most commonly affected organ in disseminated CSD. Besides ocular involvement, hepatosplenic disease (splenomegaly, splenic abscess, or granulomatous hepatitis), encephalitis, pneumonia, or osteomyelitis may be observed.^4,5^ The clinical manifestations of ocular involvement include Parinaud oculoglandular syndrome, neuroretinitis, choroidal mass, retinal infiltrate, choroiditis, branch retinal vessel occlusion, serous retinal detachment, intermediate uveitis, acute endophthalmitis, and anterior uveitis.^6,7^

This study was conducted with the aim of evaluating the various clinical findings associated with ocular involvement of CSD as well as management and follow-up of the disease.

## MATERIALS AND METHODS

The medical records of 6 patients treated and followed at the İstanbul University Faculty of Medicine, Department of Ophthalmology, Uveitis Clinic and 4 patients treated and followed at the Marmara University Faculty of Medicine, Department of Ophthalmology, Uveitis Clinic for CSD with ocular involvement between January 2007 and January 2016 were analyzed. The study was a retrospective observational case series and was conducted in accordance with the Declaration of Helsinki (2008).

The patients’ files were evaluated in terms of demographic data, history of cat contact, medical and ocular history, visual acuity, intraocular pressure (IOP) and available anterior chamber flare measurements (Kowa Company Ltd., Electronics and Optics Division, Tokyo, Japan), anterior and posterior segment findings, laboratory findings, and treatment methods used.

*The Standardization of Uveitis Nomenclature* criteria were used in the evaluation of anterior chamber and vitreous cells.^8^ We also evaluated color fundus photographs (Carl Zeiss Meditec, Hennigsdorf, Germany) taken at presentation and during follow-up, and any available fundus fluorescein angiography (Heidelberg Engineering, Heidelberg, Germany or Carl Zeiss Meditec, Hennigsdorf, Germany), optical coherence tomography (OCT) (Heidelberg Engineering, Heidelberg, Germany or OCT 3, Stratus OCT; Carl Zeiss Meditec), and 30-2 computerized perimeter (Humphrey Systems, Inc., Dublin, CA, USA) findings.

## RESULTS

Thirteen eyes of 10 patients (7 female, 3 male) with ocular CSD were included in the study. The mean age at presentation was 26.9±18.5 (6-58) years. There were 5 pediatric patients (≤16 years old). None of the patients were immunodeficient or had other systemic diseases such as diabetes. Prior to presentation, 2 patients (patients 3 and 7) had been previously misdiagnosed with noninfectious optic neuritis and treated with pulse methylprednisolone therapy without additional antibiotic therapy, while 1 patient (patient 9) had been misdiagnosed with autoimmune uveitis and treated with systemic corticosteroid monotherapy. All patients presented for ocular symptoms, and history of cat contact and systemic symptoms were only expressed upon detailed questioning. The patients’ CSD-related systemic complaints and findings prior to presentation and ocular findings at time of presentation are presented in Table 1. Nine patients had history of cat contact and had experienced symptoms indicating disseminated disease (fever, abdominal pain, weight loss, malaise, shortness of breath, and/or flu-like symptoms) starting 2-90 days earlier. For one child (patient 2), it was not clear after questioning whether or not there was a history of cat contact.

**Figure 1:**
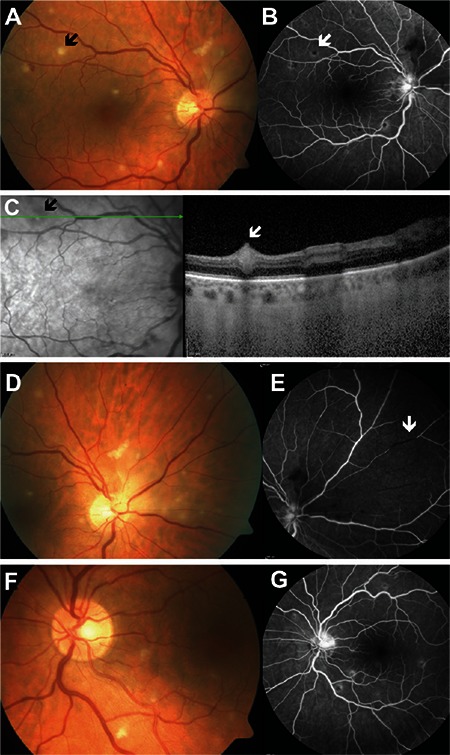
Imaging of patient 4 performed at time of presentation: right eye color fundus photographs (A and D), right eye fluorescein angiography (B and E), optical coherence tomography cross-section including retinal infiltrates in the superotemporal quadrant of the right eye (C), color photography of left eye (F), and fluorescein angiography image (G). Color fundus photography of the right eye shows multiple retinal infiltrates in the posterior pole and superonasal quadrant, and a superonasal area of retinal edema adjacent to the optic disc (A and D). Fluorescein angiography of the right eye shows partial staining of the optic disc, posterior pole retinal infiltrates with central hypofluorescence surrounded by hyperfluorescence, an area of retinal ischemia adjacent to the optic disc and arteriole filling defect (arrow) in the superonasal quadrant (B and E). Optical coherence tomography corresponding to the retinal infiltrates in the superotemporal quadrant of the right eye (indicated by arrows in A, B and C) shows focal hyperreflective retinal thickening (C). Color fundus photography of the left eye revealed multiple retinal infiltrates at the posterior pole (F). Fluorescein angiography of the left eye shows partial staining of the optic disc and posterior pole retinal infiltrates with central hypofluorescence surrounded by hyperfluorescence (G)

**Figure 2:**
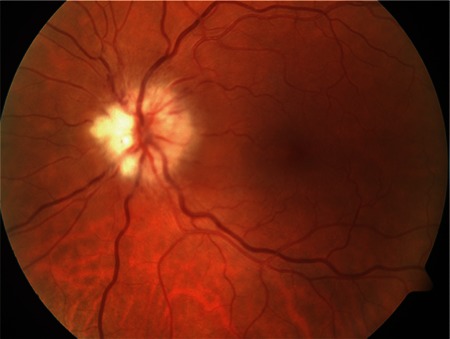
Left eye color fundus photograph of patient 6 taken at presentation shows papillitis and infiltrates in the nasal aspect of the optic disc

**Figure 3:**
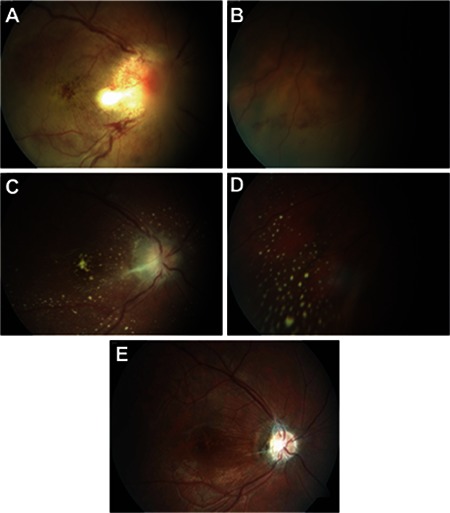
Right eye color fundus photographs from patient 7 taken at presentation (A and B), in the 4th week of treatment (C and D) and at final examination (E). Neuroretinitis, posterior pole hemorrhages, and posterior pole and inferior peripheral serous detachment are evident at presentation (A and B). Reduced optic disc edema, regression and slight pallor of the infiltrates, and multiple hard exudates in the posterior pole and inferior periphery are apparent after 4 weeks of treatment (C and D). At final examination, optic disc pallor and surrounding gliotic membrane as well as a large nerve fiber layer defect in the posterior pole are visible (E)

The patients’ ophthalmologic examination findings at presentation are summarized in Table 2. These findings included neuroretinitis in 4 eyes (associated with inferior peripheral serous retinal detachment in 1 eye), optic neuropathy in 2 eyes (1 with papillitis and optic disc infiltration, 1 with optic neuritis), retinal infiltrate in 6 eyes, retinochoroiditis in 1 eye, branch retinal artery occlusion in 3 eyes, and endophthalmitis in 1 eye (Table 1). Fundus photographs of patients 4, 6, and 7 are shown in Figures 1, 2, and 3, respectively. Visual acuity at presentation was 1.0 in 7 eyes, 0.3 in 1 eye, ≤0.1 in 4 eyes, and light perception in 1 eye. Slit-lamp examination revealed anterior chamber reaction in 2 eyes (patients 7 and 9); patient 9 also presented with endophthalmitis and exhibited wide posterior synechia and vascularized inflammatory membrane posterior to the lens in addition to anterior chamber reaction. The mean IOP of 12 of the eyes was 12.9±1.8 mmHg and mean flare value in the 7 eyes measured was 4.4±0.9 photon/ms. The eye with endophthalmitis (patient 9) was so hypotonic that IOP could not be measured by applanation tonometer. One eye that presented with retinal infiltrate (patient 3) developed branch occlusion in the infiltrated artery on the 9^th^ day of treatment.

All patients tested positive for *Bartonella henselae* immunoglobulin (Ig) M and/or IgG. The results of detailed laboratory, systemic, and ocular imaging are summarized in Table 3. Diagnostic vitreal aspiration was performed on the eye with endophthalmitis (patient 9), but no bacteria, fungi, or hyphae were visible on direct inspection. Bacterial and fungal cultures were negative. The vitreal fluid was determined acellular by cytopathologic analysis.

All patients received antibiotic (doxycycline, ciprofloxacin, clarithromycin, azithromycin, rifampicin, ceftriaxone) therapy. This therapy was augmented with intravenous pulse methylprednisolone therapy for 5 days in 2 patients and 15-40 mg/day oral corticosteroid therapy in 4 patients. Antibiotic therapies and systemic corticosteroid doses and durations administered to the patients are shown in Table 4. Treatment was ongoing for patient 6; the mean treatment duration for the other 9 patients was 47±14.5 (21-63) days. Table 5 shows the patients’ ophthalmologic examination findings at final examination. Final visual acuity was 1.0 in 9 eyes, 0.8 in 1 eye, 0.4 in 1 eye, and no light perception in the eye that presented with endophthalmitis. Mean follow-up time was 106±79.7 (21-270) days.

## DISCUSSION

CSD is a zoonotic disease which shows no discrimination based on gender or race. Though it may occur in patients of any age, the large majority of reported cases are in children and adolescents. According to the literature, adults represent an average of 20% of cases; however, 50% of the cases in our study were adults.^9^

*Bartonella henselae* often causes chronic bacteremia in kittens and nursing cats, and previous studies have reported feline infection rates of 10-40%.^1,2^ Studies have also shown that 90-95% of CSD patients have a history of cat contact, although ocular CSD has also been documented in patients without a history of cat contact.^10^ Nearly all of the patients in our series reported cat contact. However, patients only offered specific information regarding cat contact and systemic complaints when asked. None of the patients had been previously diagnosed with CSD, even those who had seen a doctor for the systemic symptoms they experienced prior to their ocular complaints. Therefore, raising awareness of the ocular findings of CSD is important in terms of diagnosis.

The most common and classic sign of ocular CSD is neuroretinitis characterized by sudden, painless vision loss, but this sign is not pathognomonic. Although *Bartonella henselae* is identified as the etiologic factor in two-thirds of neuroretinitis cases, it can also be caused by Behçet’s disease, toxoplasma and other infectious diseases.^11,12^ Neuroretinitis is usually unilateral, though bilateral cases have also been reported.^13^ Visual acuity in the affected eye can vary between light perception and 1.0 at presentation and vision may rapidly deteriorate within a matter of days. Patients often exhibit relative afferent pupillary defect, dyschromatopsia, and central, cecocentral, or arcuate visual field defects. Macular star may appear a few days after vision loss begins and become more distinct over 2-3 weeks.^11,12,13^ In our case series, we noted isolated unilateral neuroretinitis in 2 patients, neuroretinitis with serous retinal detachment in the inferior quadrant in 1 patient, and unilateral neuroretinitis with contralateral retinal infiltrate and subsequent inferotemporal branch arteriolar occlusion in 1 patient. Despite the presence of atypical findings accompanying neuroretinitis in these 2 patients, they had been diagnosed as optic neuritis and treated with pulse methylprednisolone at other medical centers. Isolated optic neuritis may occur rarely in CSD. In our case series, isolated optic neuritis was only observed in one child. Particularly in children and young adults, infectious agents like *Bartonella henselae* must be excluded before initiating pulse methylprednisolone therapy for neuroretinitis or optic neuritis.

CSD may also clinically manifest with retinal infiltrates resembling cotton-wool exudates, retinochoroiditis, retinal artery occlusion, or endophthalmitis, as we observed in our case series. Superficial infiltrates appearing as soft exudates lacking vitreous cells were observed on the retinal surface in 4 patients and on the optic disc in 1 patient in our series. Although the mechanism by which these infiltrates form is not fully understood, it is believed they arise secondary to ischemia resulting from retinal arteriole occlusion.^14^ The superficial retinal infiltrates seen in ocular CSD must be differentiated from retinitis or retinal infiltrates seen in ocular manifestations of Behçet’s uveitis, sarcoidosis, rickettsia and toxoplasma. Retinal infiltrates in CSD show central hypofluorescence and surrounding hyperfluorescence on FA. On OCT, they appear as focal hyperreflective thickening, particularly in the inner retinal layers. This OCT finding resembles the retinal infiltrates seen in Behçet’s and rickettsia. Typical OCT findings in toxoplasma retinochoroiditis are focal choroidal thickening under the lesion and concentrated cell infiltration in the posterior hyaloid overlying the lesion. The retinal infiltrates seen in active Behçet’s uveitis are generally accompanied by diffuse vitritis, whereas vitreous cells are usually not present over CSD retinal infiltrates.

Superficial retinal infiltrates associated with CSD require close follow-up, as they can lead to branch artery thrombosis, as we observed in our cases. *Bartonella henselae* is an intracellular bacterium that infects erythrocytes and endothelial cells, and may cause vascular occlusion due to its affinity for vascular endothelium. Branch retinal artery occlusion due to CSD has been documented in the literature in various case reports and series.^15,16,17,18,19,20,21^ Patients exhibit alterations in visual acuity in accordance with the location of the affected artery. Because our patient’s peripheral arteries were involved, his central vision was unaffected, but there was permanent visual field loss in the area corresponding to the occlusion.

Endophthalmitis is a rare presentation of CSD, and only a few such cases have been previously reported. In these patients, Bartonella serology may yield negative results from serum but positive results from vitreous fluid.^22^ In our case, serum was positive for *Bartonella henselae* IgM and IgG, thus eliminating the need to evaluate the vitreous fluid.

CSD may cause severe systemic involvement in immunosuppressed patients. It has been reported to lead to bacillary angiomatosis in patients who are HIV positive.^23^ None of our patients were immunocompromised and none exhibited any signs of angiomatosis. On the other hand, we have never encountered ocular CSD in any of the HIV-positive patients followed in our clinic.

CSD is diagnosed based on clinical (systemic and/or ophthalmologic) symptoms and findings; serologic tests support the diagnosis. High *B. henselae* IgM titer is an indicator of acute infection and values typically return to normal within 3 months. *B. henselae* IgG rises over time and remains positive up to 2 years. Positive *B. henselae* IgM or high *B. henselae* IgG titer are sufficient for CSD diagnosis.^24^ In the present study, all patients tested positive for *B. henselae* IgM and/or IgG. Five patients were positive for both IgM and IgG, 4 were positive for just IgG, and 1 was positive only for IgM.

CSD is self-limited in individuals with healthy immune systems, and treatment is controversial. Treatment with erythromycin, doxycycline, or azithromycin is recommended for patients without immune deficiency or other systemic diseases like diabetes. Rifampicin, trimethoprim-sulfametoxazol, quinolones, or intravenous aminoglycosides are also effective treatment alternatives.^25,26^ Of the cases in our series, doxycycline was the most commonly used antibiotic and in most cases was administered in combination with quinolone, macrolide, and/or rifampicin. The duration of antibiotic treatment is disputed. HIV-positive patients are recommended to continue treatment for 2 to 4 months, whereas 10 days to 3 weeks has been reported as sufficient for patients with ocular involvement.^4^ The use of systemic corticosteroids in treatment is also a subject of debate. In the present study, we were unable to assess the effect of systemic corticosteroids on prognosis because the cases in our series represented the treatment approaches of two different clinics, the number of patients was low, and the study was retrospective. The prognosis was very good in all patients except the case with endophthalmitis.

## CONCLUSION

CSD is not limited to neuroretinitis or optic neuritis, but can also manifest with superficial retinal infiltrates, retinal artery occlusion, or endophthalmitis. Asking patients about their history of cat contact and performing Bartonella henselae serologic analysis are important in the differential diagnosis of these clinical manifestations.

### Ethics

Ethics Committee Approval: The research followed the tenets of the Declaration of Helsinki, Informed Consent: An informed consent was obtained before all diagnostic and therapeutic procedures.

Peer-review: Externally and Internally peer-reviewed.

## Figures and Tables

**Table 1:**
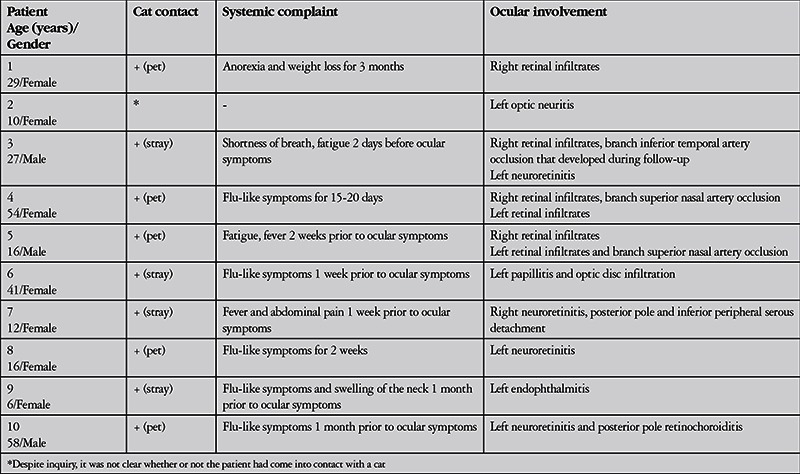
Previous systemic complaints and ocular examination findings at time of presentation in patients with ocular involvement of cat scratch disease

**Table 2:**
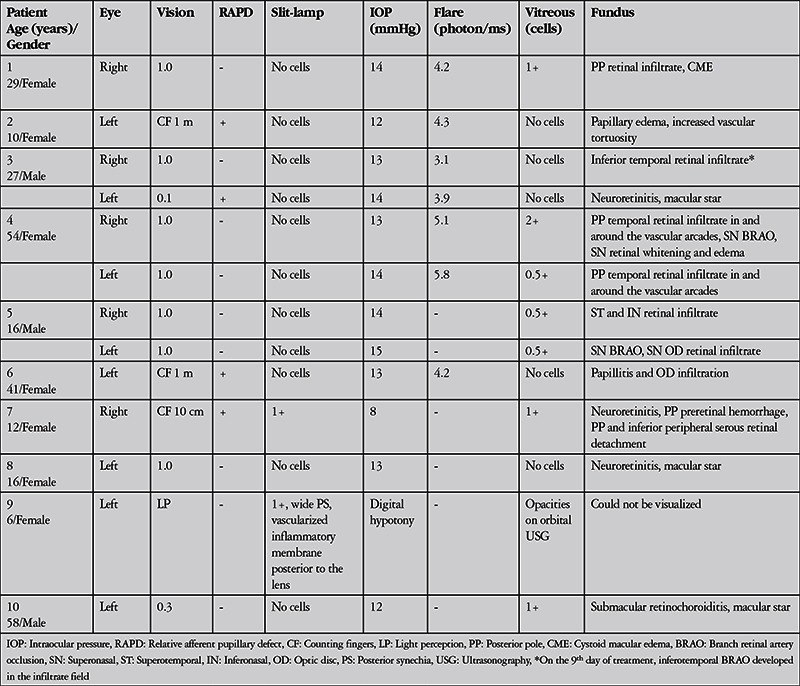
Ocular examination findings at presentation in patients with ocular involvement of cat scratch disease

**Table 3:**
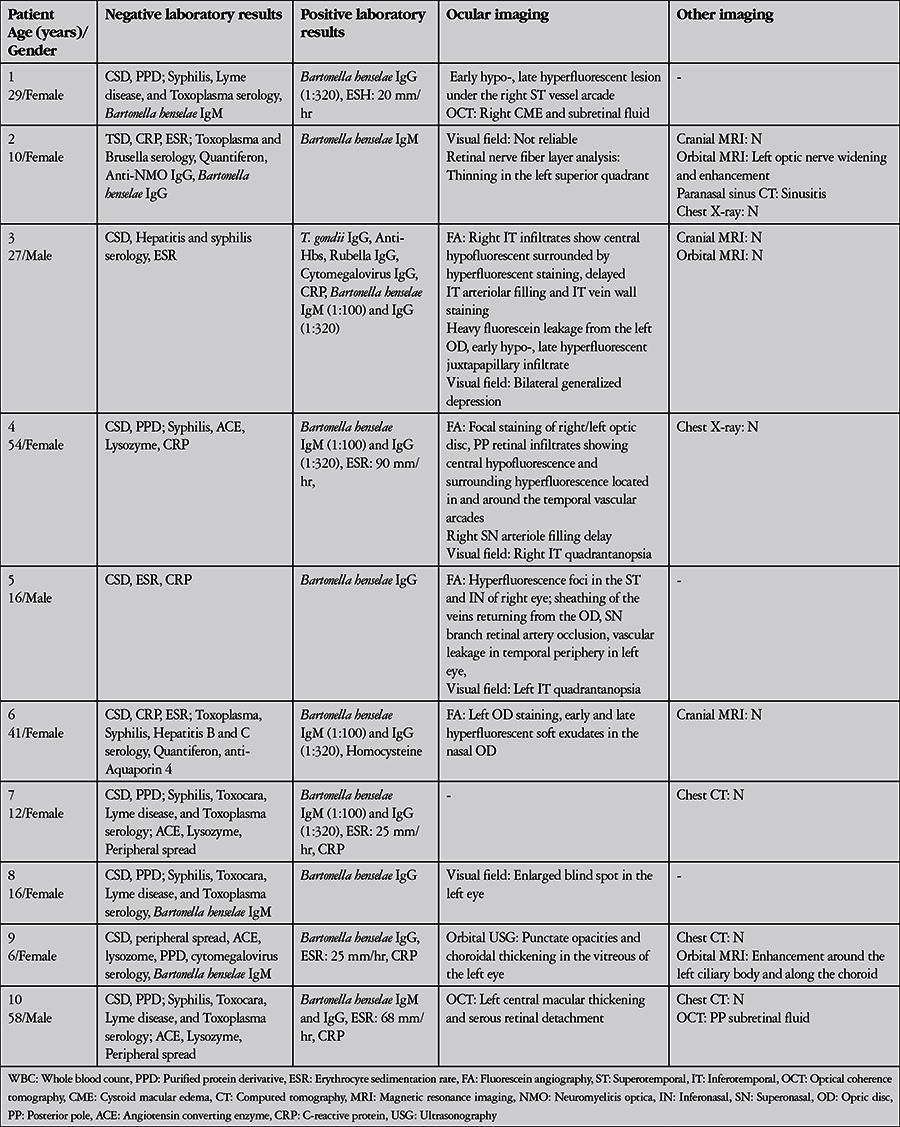
Laboratory findings and imaging results at presentation in patients with ocular involvement of cat scratch disease

**Table 4:**
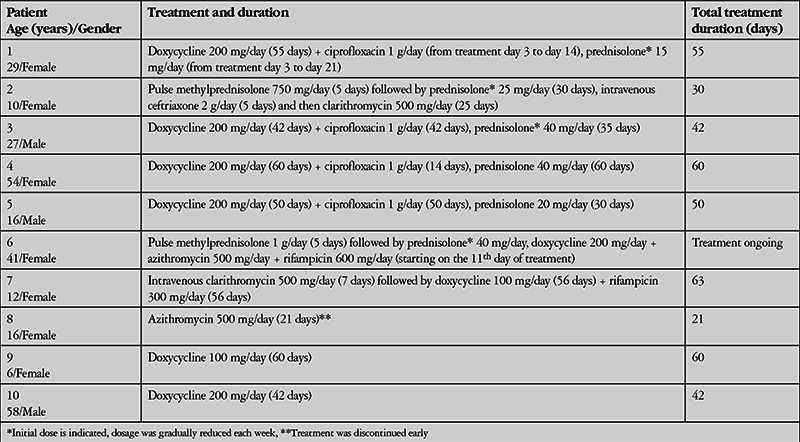
Treatment methods and durations in patients with ocular involvement of cat scratch disease

**Table 5:**
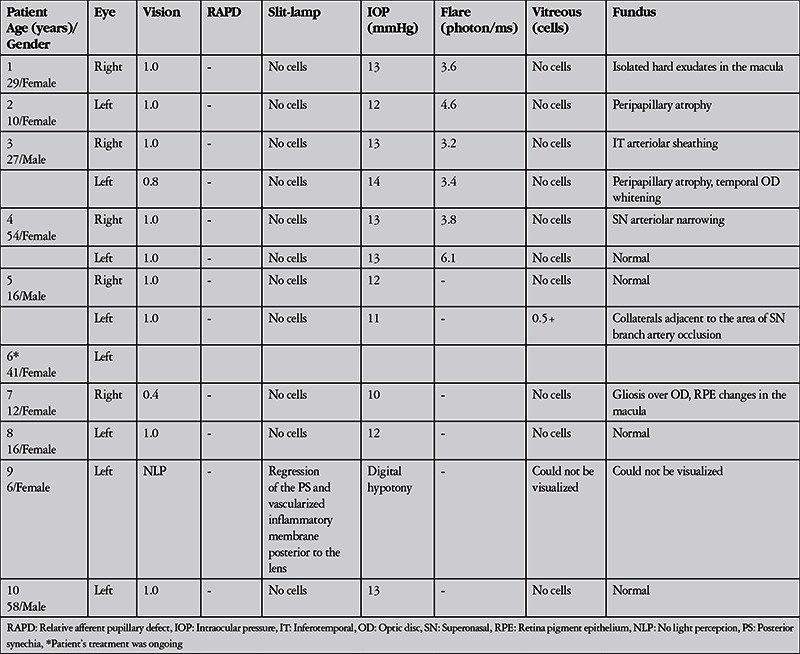
Ocular examination findings at final follow-up in patients with ocular involvement of cat scratch disease
